# Bacterial dominance is due to effective utilisation of secondary metabolites produced by competitors

**DOI:** 10.1038/s41598-020-59048-6

**Published:** 2020-02-11

**Authors:** Benjamin G. Morgan, Paul Warren, Ryan E. Mewis, Damian W. Rivett

**Affiliations:** Department of Natural Sciences, School of Science and the Environment, Manchester Metropolitan University, Manchester, UK

**Keywords:** Microbial ecology, Clinical microbiology

## Abstract

Interactions between bacteria govern the progression of respiratory infections; however, the mechanisms underpinning these interactions are still unclear. Understanding how a bacterial species comes to dominate infectious communities associated with respiratory infections has direct relevance to treatment. In this study, *Burkholderia, Pseudomonas*, and *Staphylococcus* species were isolated from the sputum of an individual with Cystic Fibrosis and assembled in a fully factorial design to create simple microcosms. Measurements of growth and habitat modification were recorded over time, the later using proton Nuclear Magnetic Resonance spectra. The results showed interactions between the bacteria became increasingly neutral over time. Concurrently, the bacteria significantly altered their ability to modify the environment, with *Pseudomonas* able to utilise secondary metabolites produced by the other two isolates, whereas the reverse was not observed. This study indicates the importance of including data about the habitat modification of a community, to better elucidate the mechanisms of bacterial interactions.

## Introduction

Bacterial community dynamics rely on a complex set of interactions with the biological and environmental factors of an ecosystem^1^. These interactions dictate the structure of the community and, therefore, the functioning of the ecosystem. Interactions are also fundamental in maintaining, or mitigating dominance of fast-growing bacteria, especially over time due to depletion of nutrients or the changing balance of competitive/cooperative interactions^2^. This mitigation of dominance from fast-growing bacteria can be undermined if there are selection pressures conferring a fitness advantage to the dominant population, or if the other members of the community actively support the growth of the fast grower^3^.

Considerations as to the mechanisms by which a single bacterial population can come to dominate a community is of particular importance in infection microbiology^4–6^. Chronic infections, as are found in the respiratory tracts of individuals suffering from chronic obstructive pulmonary disease or cystic fibrosis (CF), are increasingly being characterised as a polymicrobial infection^7–9^. Despite this, one of the clinically important metrics in the prognosis of CF-associated lung disease remains the detection of *Pseudomonas aeruginosa*; if this bacterial species is cultured then there is a high likelihood of a worsening of the patients’ respiratory function^10^. Further, using molecular methods, researchers have observed that *P. aeruginosa* dominates the infectious community in CF in terms of both relative and absolute abundance, however, the mechanism for this is unclear^4,5^.

A previous study, screened a number of *P. aeruginosa* isolates to assess whether they could be discriminated by their ability to alter a synthetic lung environment *in vitro*^11^. To identify the differences, the authors employed proton nuclear magnetic resonance (^1^H NMR), which detects changes in chemical structures that contain hydrogen atoms^12^. Whilst this study identified a link between pathogenicity and environment manipulation, there was no consideration to how this habitat changes influences other bacteria.

The way in which bacteria modify their environment, can have a direct influence on the nature of the biotic interactions (e.g. reduced or increased competition^2,13,14^), due to changing resource utilisation, and the diversity of the system, through changing selection pressures (e.g. bioremediation of a toxin^15^). As such, one study also utilised ^1^H NMR to look at the effect of shared resources, and complimentary interactions (e.g. cross-feeding) in a recalcitrant, minimal media^16^. The authors of this study found that complementary interactions increased through diversification of resources, which was corroborated by subsequent study^2^. Further, continued study has indicated that there is a strong positive biodiversity-ecosystem function relationship (i.e. as the number of species within a system increases, the measure of functioning does also) in species-rich bacterial systems^2,17–19^. How environment changes affect bacteria during respiratory infections has not been studied previously.

Our study was designed to test the hypothesis that the modification of an environment by *P. aeruginosa* would negatively affect the growth of other bacteria within the community. We approached this using simple, manipulated microcosms containing three bacterial populations implicated in different stages of CF-associated lung disease^20^; *P. aeruginosa* is regarded as an terminal coloniser, in-so-far-as this species is associated with end-stage disease^21^, *Staphylococcus aureus* is commonly identified during paediatric samples, and is known to be excluded by the presence of *P. aeruginosa*^22^, and *Burkholderia cepacia*, which can be identified at all stages of lung infection, with differing outcomes dependent on the patient^23^. By using a simple microcosm system and detailed habitat profiling (^1^H NMR) we will elucidate the mechanisms by which the bacteria interact and affect changes in community dominance.

## Results and Discussion

### Microbial interactions are dependent on diversity and time since inoculation

**Figure 1:**
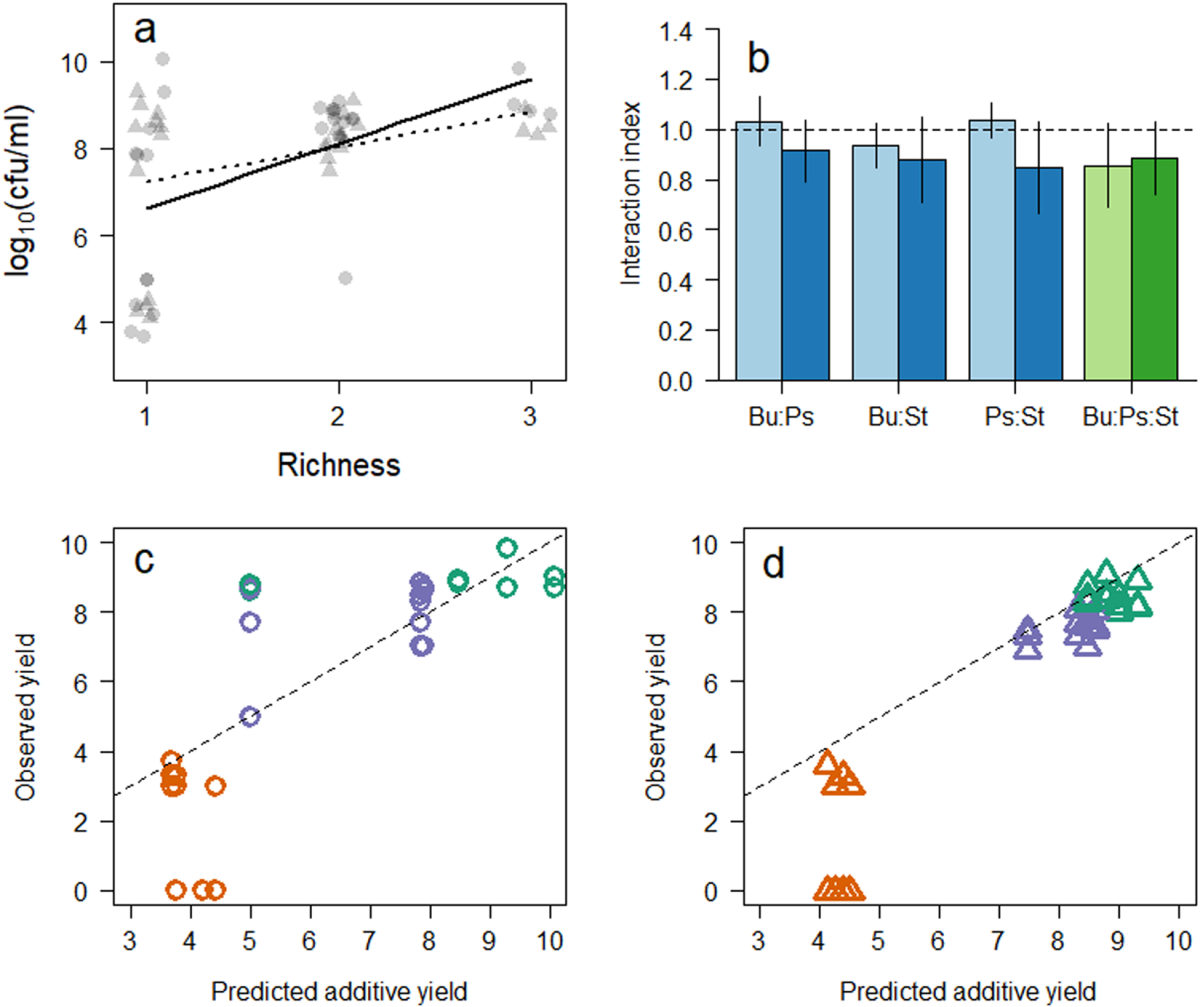
Changes in bacterial growth and media due to composition and time. (**a**) Biodiversity ecosystem function plot of bacterial biomass against microcosm richness after 48 (circles) and 168 (triangles) hour incubation. Relationship is significant after 48 (solid line) but not after 168 hours (dotted line). (**b**) Mean (±standard deviation) interaction index, calculated as a ratio of observed to expected bacterial yield, where values > 1 represents positive interactions, 1 no interaction (dashed line), and <1 negative interactions. Blue bars represent two species co-cultures, green bars are three species co-cultures. Observed to predicted yields of the individual isolates after (**c**) 48 and (**d**) 168 hours incubation, where green, orange and purple points represent *Pseudomonas, Burkholderia* and *Staphylococcus* isolates respectively.

The link between increasing biodiversity and primary productivity is well-established in environmental bacteria, and our results confirmed this relationship in clinical isolates (Fig. 1a) was significant (β_48_ = 1.49 ± 0.46 log_10_(colony forming units (cfu) ml^−1^) species^−1^, R^2^ = 0.29, p = 0.003) over time scales encompassing initial growth, but became non-significant (β_168_ = 0.79 ± 0.39 log_10_(cfu ml^−1^) species^−1^, R^2^ = 0.14, p = 0.051) as the community established. This indicated that the influence of increasing diversity reduces over time, which is thought to be a function of changing resource availability. If we consider these positive biodiversity-productivity relationships *in situ*, then it would be expected that the more diverse a community, within a patient’s respiratory tract, the greater the bacterial load. Research suggests, however, that this is not the case, with many patients having constant bacterial loads^5^. This is likely due to the disparate regions of the respiratory tract^24^. The interactions indicated in this study are therefore most likely localised to pockets within the lung as seen with other, structurally complex environments^25^. This could be a cause of localised airway damage, through recruitment of neutrophils^26^, but further work is needed to understand the implications of these ecological observations.

To understand the impact changing combinations had on the growth of the bacteria in the microcosms, we predicted the primary productivity of the microcosms based on the productivity of the monocultures (assuming no interaction so the growth would be additive^18,27^) and compared to the observed productivity^2,18,28^. Here, values >1 represents positive interactions, 1 no interaction, and <1 negative interactions^18^. We found a significant difference (F_1,27_ = 7.84, p = 0.009) in mean change (mean ± 1 s.e. throughout) in abundance (48 hours: −0.44 ± 0.47; 168 hours: −1.88 ± 0.42 log_10_ (cfu ml^−1^)), suggesting that antagonism increases the longer the bacteria cohabited (Fig. 1b). This is in contrast to previous studies that indicate the reduction in antagonistic interactions over time, due to niche differentiation^2^. We therefore postulated that this increase in apparent competition was due to a direct exclusion of one bacteria by another.

To test this postulation, the effects of each isolate on the others in co-culture was monitored by calculating the difference in abundance change from the predicted productivity for each isolate individually. Our results indicated that there was no significant change in abundance for the *Pseudomonas* (1.19 ± 0.004, One-sample Wilcoxon rank test (µ = 1) p = 0.783) and *Staphylococcus* (1.15 ± 0.004, p = 0.142) isolates, whereas the *Burkholderia* isolate performed significantly worse (0.46 ± 0.031, p = 0.003) when in co-cultures after 48 hours incubation (Fig. 1c). After 168 hours, all the isolates had significantly reduced in their mean observed to predicted ratio (Fig. 1d); *Burkholderia* (0.25 ± 0.037, p = 0.002) *Pseudomonas* (0.95 ± 0.030, p = 0.016), *Staphylococcus* (0.92 ± 0.021, p = 0.004). This result supported our postulation that the reduction in overall ecosystem function was due to the exclusion of the *Burkholderia* isolate by the other bacteria. With the removal of this isolate from many of the microcosms, the *Pseudomonas* and *Staphylococcus* isolates may have expended energy out-competing the *Burkholderia*, thereby losing any potential synergistic benefit. Whilst we observed no evidence as to the direct effects of inhibitory second metabolites there is evidence that specific antagonistic interactions do occur in similar microcosm experiments^22^. It is therefore possible that specific, non-resource mediated interactions caused the apparent extinction (isolate fell below the detection limit of 50 cfu ml^−1^), as previously observed in co-culture experiments^29^. There has been no study that investigates the relative contributions of different mechanisms of competition (i.e. ecological or chemical) despite much literature stressing the interplay^30–33^, however, there are an increasing number of studies^22,27,29,34–36^, including this one, where this holistic approach would be greatly informative.

### Niche overlap

**Figure 2:**
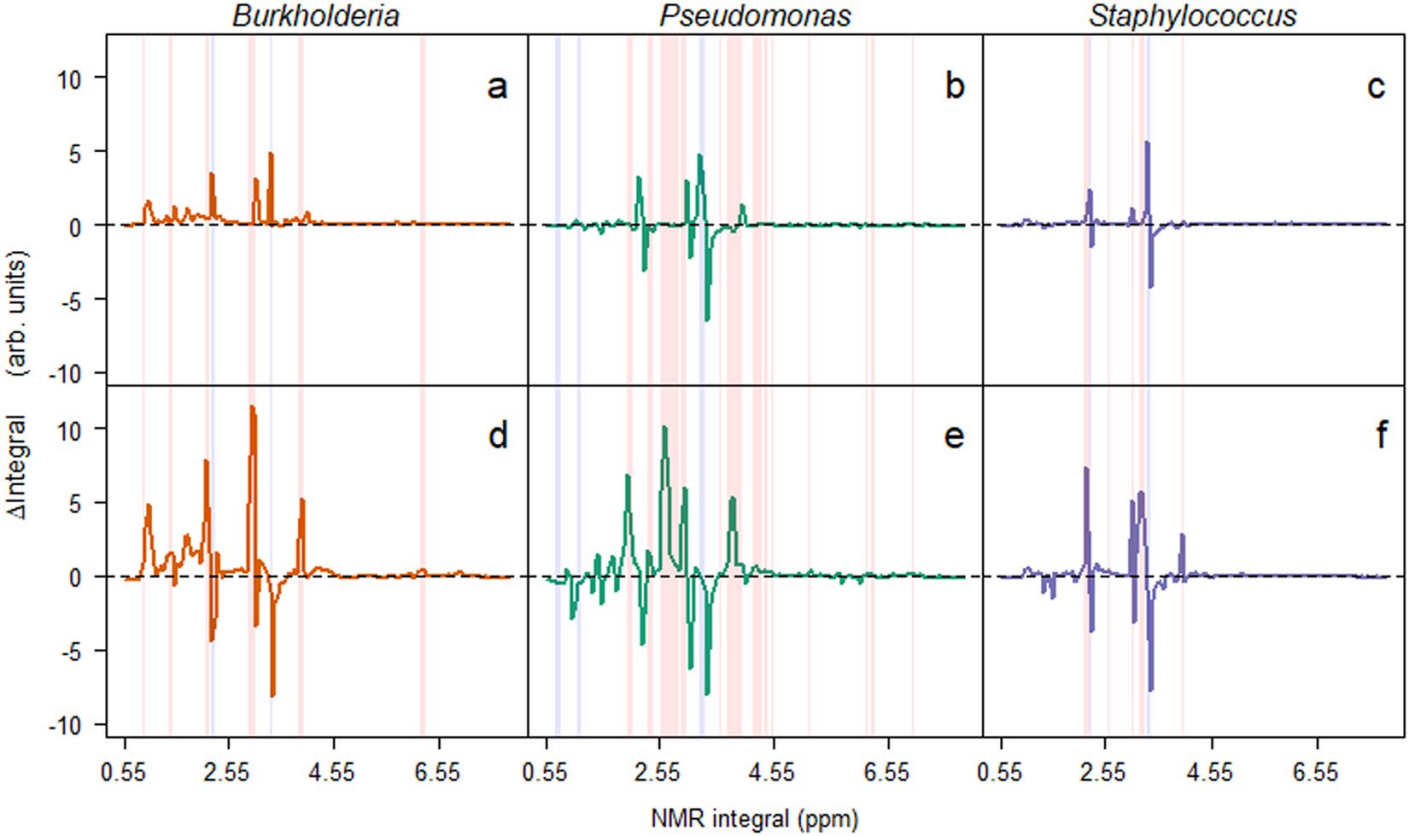
Bacterial induced changes in ^1^H NMR integrals for microcosms at the two time points. Changes in integrals (Δ integrals) were calculated by subtracting integrals for the no bacteria control samples from the integrals recorded from microcosm media from microcosms containing bacteria in monoculture. Mean Δ integrals are plotted against the location (ppm) of the integral. Red and blue columns represent integrals that significantly increase and decrease, respectively, from the first (**a**–**c**) to second time point (**d**–**f**).

Having found that the *Burkholderia* isolate was excluded by the other two isolates, we used ^1^H NMR to understand how this was possible, and tested the mechanism of competition as either due to competitive exclusion by removal of nutrients or through the production of toxic secondary metabolites. By taking integrals of the ^1^H NMR spectra at 0.05 ppm intervals (Supplementary Figs. S1 and S2), and calculating the differences between the monocultures and the blank samples we could identify relative changes in the media. At the first time points (Fig. 2a–c), these differences in integrals indicated that changes were predominantly (62.56%, n = 274) positive (creation of new metabolite), with the remainder (37.44%, n = 164) of integrals decreasing (degradation of nutrient) in intensity. Over time, the number of positive and negative integrals became equivalent; 50.91% (n = 223) positive and 49.09% (n = 215) negative integrals (Fig. 2d–f). Using these data, the niche overlap was calculated between the isolates. There was a significant (χ^2^_3_ = 30.38, p < 0.001) increase in overlap in the latter time point across the combinations, suggesting that the bacteria were competing for the same resources. This may have been due to the low diversity media (carbon sources were almost entirely amino acids^11^) the bacteria were grown in, therefore the opportunity for niche diversification was limited. This increase in overlap was strongly, albeit non-significantly, correlated (*Rho* = −0.619, p = 0.115) with a reduction in the mean observed to predicted ratio in each of the microcosms (Supplementary Fig. S3). Interestingly, the *Pseudomonas* isolate was observed to have a greater number of negative integrals than either of the *Burkholderia* and *Staphylococcus* isolates. This is likely due to *P. aeruginosa* being an generalist through its ability to adapt rapidly to the lung environment^37^. With the increasing overlap in resource utilisation, this is an example of competitive exclusion in bacteria^38^, and further explains the extinction of the *Burkholderia* isolate.

### Changes in environment are indicative of community structure

**Figure 3:**
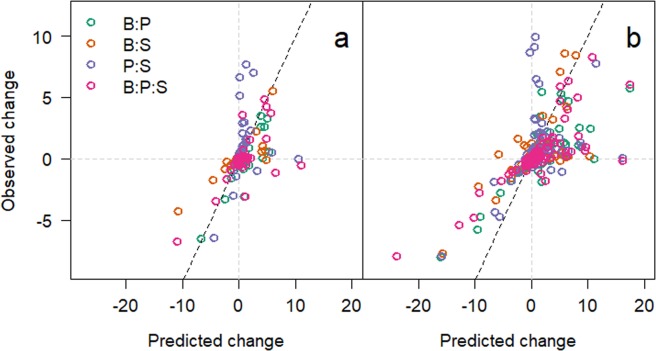
(**a**) Interaction index of each ^1^H NMR integral for the medium in each microcosm at 48 hours. Green circles are integrals taken from microcosms with *Burkholderia* and *Pseudomonas*, orange for *Burkholderia* and *Staphylococcus*, purple for *Pseudomonas* and *Staphylococcus*, and pink for all three species together. (**b**) Interaction index for 168 hours incubation. Grey dotted lines indicate the zero lines with the dotted black line showing the relationship if there were no interactions (1:1) for comparison.

To understand the interplay between environmental modification and changes in bacterial interactions, we plotted the interactive index (predicted/observed)^18^ using the integrals measured in microcosms with monocultures added together as the predicted. This indicated that as the isolates grew, the number of significant interactions (change in integral > mean change ± 2 s.d.) increased significantly (χ^2^_3_ = 32.39, p < 0.001) from 48 (Fig. 3a) to 168 hours (Fig. 3b).

**Figure 4:**
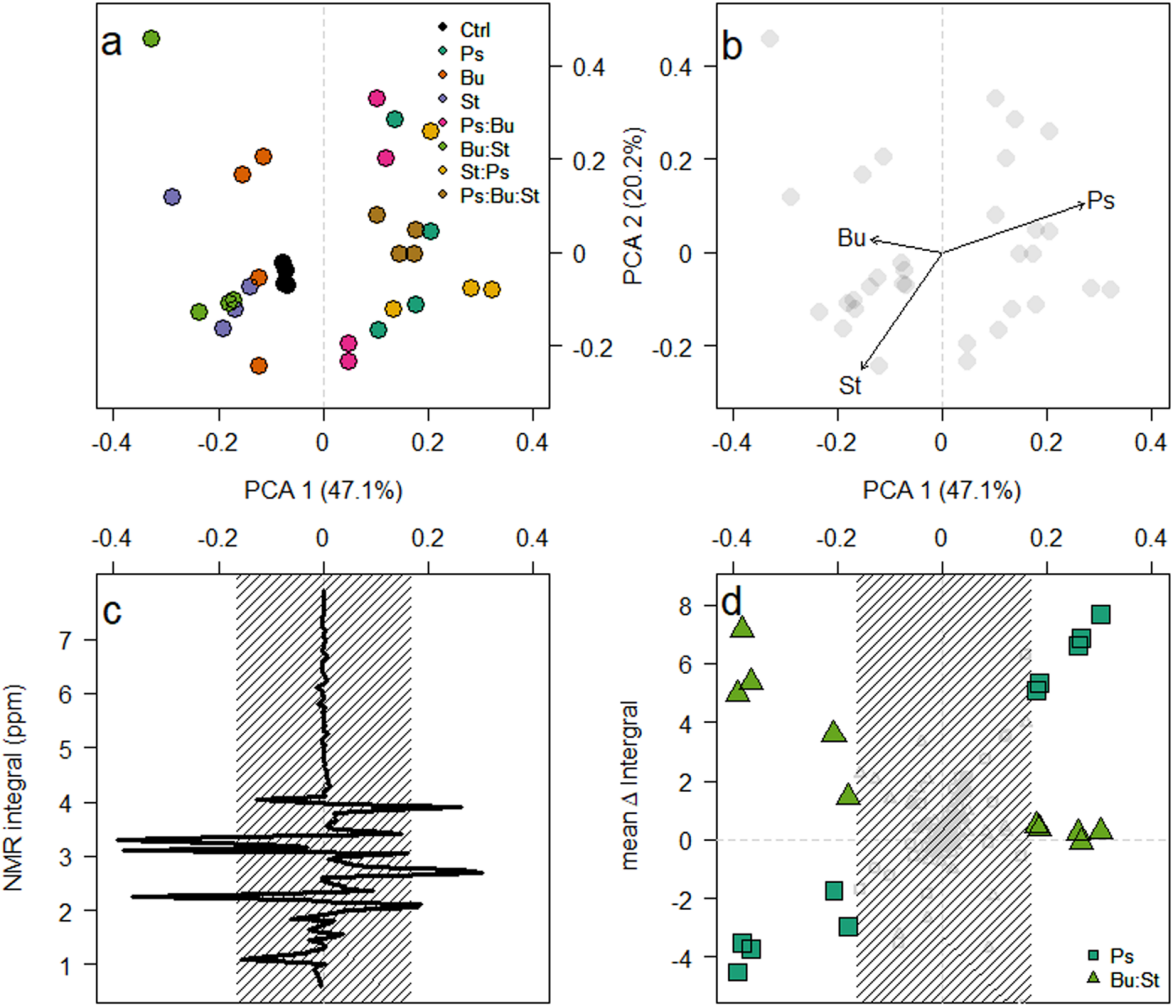
The modification of media is dependent on the composition of the microcosm. (**a**) PCA ordination plot of the difference in ^1^H NMR integrals between sampling points. (**b**) Correlations between the abundance of each isolate at the final time point was assessed and the shift in PCA1 was indicated to be due to the growth of *Pseudomonas*. (**c**) Loading plot of PCA1 against the ^1^H NMR integrals. Peaks were deemed significant if they lie outside the shaded region (mean ± 2 standard deviations). Finally, (**d**) indicates the mean change in integral for microcosms that contain the *Pseudomonas* isolate (squares) and those that do not (triangles).

To demonstrate the differences between samples, the readings from the first were subtracted from the second time point. The results indicated that the primary Principle Component Analysis (PCA) clustering (Fig. 4a) was based on the abundance of *Pseudomonas* (R^2^ = 0.32, p = 0.006) and *Staphylococcus* (R^2^ = 0.21, p = 0.023) at the final time point, but not *Burkholderia* (R^2^ = 0.08, p = 0.314)(Fig. 4b). The primary PCA axis was primarily correlated to the abundance, or absence, of the *Pseudomonas* isolate within a microcosm (Fig. 4b), and was determined by changes in 10 integrals (Fig. 4c). The mean change in integral was plotted against the primary PCA axis for microcosms depending on *Pseudomonas* presence (Fig. 4d). For microcosms containing *Pseudomonas*, these values had a significant positive relationship (β = 16.91 ± 0.82, F_1,145_ = 419.53, p < 0.001); conversely, those microcosms where *Pseudomonas* was absent had a significant negative relationship (β = −8.55 ± 1.08, F_1,145_ = 62.96, p < 0.001). This detailed investigation of the ^1^H NMR profiles suggests a potential mechanism that facilitates the domination of *Pseudomonas*, as evident in these microcosms. Whilst we cannot exclude the possibility of the secretion of inhibitory molecules (e.g.^29^), our data highlight the ability of the *Pseudomonas* isolate to utilise secondary metabolites created by other bacteria. We do not observe a reciprocal relationship between the *Burkholderia* and *Staphylococcus* isolates and metabolites produced by the *Pseudomonas* isolate. Putative identification of the ^1^H NMR peaks, suggest that those that significantly increase in the presence of *Pseudomonas* are derivatives of the amino acid serine, potentially the quorum sensing molecule homo-serine lactone^39^. As these data indicate an increase in the serine-associated peaks at the second time point compared with the first, this would equate to the *Pseudomonas* isolate being at stationary phase with no fresh intake in nutrients, therefore a decrease in metabolism would be beneficial^40^. Conversely, the peaks produced by the *Burkholderia* and *Staphylococcus* isolates, that are used in the microcosms with *Pseudomonas*, correspond with protons attached to a carbon atom, adjacent to an aromatic ring. There is evidence that aromatic compounds have an inhibitory effect on *Pseudomonas* species’ competitiveness^41^. Due to the evolutionary history shared by these isolates (i.e. having come from the same environment, potentially in close proximity to one another), the *Pseudomonas* isolate could have evolved to metabolise these compounds produced, as a defence mechanism, by its competitors. Further work would be required to know whether this is a true mechanism. Our data strongly suggest that cross feeding occurs between the bacteria, on an ecological timescale and not limited to evolutionary times^16^, ultimately to the advantage of the *Pseudomonas* isolate. This media was used to mimic the chemical conditions experienced in the lung environment from which these bacteria were isolated^11,42^. Previous studies have indicated that the transcriptomic expression profiles are highly similar in synthetic cystic fibrosis medium to those observed in their natural environment^43,44^. As such, we believe that this mechanism of unidirectional cross-feeding is likely to be present in natural systems and should be investigated further.

## Conclusion

Our results confirmed previous results that the influence of increasing richness weakens over time in bacterial microcosms^2^, but there was a significant positive relationship between biodiversity and cell numbers. This relationship between increasing productivity and diversity is present despite interactions between bacteria are predominantly negative. Previous bacterial microcosm experiments, using environmental isolates^2,17,19^, have supported the presence of this positive relationship despite antagonistic interactions being the most prevalent. Conversely, the only other microcosm experiment that used clinically-derived isolates with a similar design to this^28^ indicated that there was a greater level of synergistic interactions occurring. We consider that our results suggest an ecological mechanism as to how *P. aeruginosa* dominates an infectious community which is in contrast to the production of bacteriocins^45^, however, we saw no evidence of this in our study.

CF is a genetic disease, and thus affects the individual throughout their life, with a multitude of clinical interventions occurring dependent of disease severity and progression^46^. We focused our study on potential ecological interactions between some of the most clinically relevant bacteria based on different resource utilisation profiles. The potential impact of treatment regimens on the bacterial community, and resulting changes in interactions, should be the focus of subsequent studies. Previous ecological investigations based on environmental isolates have indicated that interactions are affected by abiotic stresses^47,48^, but the magnitude of the effect is predicated on the communities’ resistance, or resilience, to the perturbation^49^. In CF, antibiotic treatments can cause shifts in community structure that can influence the trajectory of the community, and the selection of resistant strains to become dominant^4,46^.

This initial study is the first to utilise ^1^H NMR to postulate mechanisms that dictate interspecies competition in restricted microcosm systems. By using this analytical technique, we were able to identify the interactions between the species based on the utilisation of the media to understand why the apparent dominance of *Pseudomonas* was observed. Isolates of *Pseudomonas* have been reported to be metabolically diverse^11,28^, which has been purported as a method to identify this genus^11^. Further studies are required to validate these findings, but the incorporation of microbe dependent environmental change is vital to assigning mechanisms to the observed ecology.

## Experimental Procedures

### Bacterial samples

Bacteria were isolated from a single sputum sample from an individual with CF attending an outpatients clinic at Southampton General Hospital, UK (Male, age = 26 years, ΔF508 homozygote, BMI = 23.7, not presenting with Diabetes or liver disease). Written informed consent was obtained from the patient for this study. The sputum sample was taken under full ethical approval from Southampton and South West Hampshire Research Ethics Committee (NHS REC number: 08/H0502/126) in accordance with relevant guidelines and regulations. The sample was stored at −80 °C after transport from the clinic to the laboratory. Isolates of *Pseudomonas aeruginosa, Burkholderia cepacia* and *Staphylococcus* sp. colonies were isolated by inoculating *Pseudomonas aeruginosa* selective agar (CM0559 with SR0102, ThermoFisher Scientific UK, Altrincham, UK), *Burkholderia cepacia* selective agar (CM0995B with SR0189E, ThermoFisher Scientific UK, Altrincham, UK) and Mannitol Salt Agar respectively (ThermoFisher Scientific UK, Altrincham, UK). Colonies were picked and purified on the appropriate selective media and stored at −80 °C in 30% v/v (final concentration) glycerol until use.

### Experimental medium

For the experiment, a synthetic CF medium (SCFM) adapted from Kozlowska *et al*.^11^ was used, briefly; 10 g L^−1^ BSA, 1.4 g L^−1^ herring sperm DNA, 5 g L^−1^ egg yolk emulsion, 51.8 mM NaCl, 14.9 mM KCl, 10 mM MOPS, 2.28 mM NH_4_Cl, 1.754 mM CaCl_2_, 1.3 mM NaH_2_PO_4_, 1.25 mM Na_2_HPO_4_, 0.606 mM MgCl_2_, 0.348 mM KNO_3_, 3.6 µM FeSO_4_, 2.128 mM L-lysine HCl, 1.78 mM L-alanine, 1.661 mM L-proline, 1.609 mM L-leucine, 1.549 mM L-glutamate HCl, 1.446 mM L-serine, 1.203mM L-glycine, 1.12 mM L-isoleucine, 1.117 mM L-valine, 1.072 mM L-threonine, 0.827 mM L-aspartate, 0.802 mM L-tyrosine, 0.676 mM L-ornithine HCl, 0.633 mM L-methionine, 0.53 mM L-phenylalanine, 0.519 mM L-histidine HCl, 0.306 mM L-arginine HCl, 0.16 mM L-cysteine HCl, 0.013 mM L-tryptophan. The medium was adjusted to a pH of 6.8 and filter sterilised (0.45 µm pore size) prior to use.

### Experimental microcosms and design

Bacterial monocultures were grown for 48 hours until they reached late exponential/early stationary phase (Supplementary Fig. S4). Each monoculture was then diluted to an optical density (λ = 600 nm) of 0.1 in fresh SCFM, and combined so that the bacterial load of each inoculum remained constant regardless of the number of species present^28^. The bacterial mixtures were inoculated 1:49 (~1 × 10^3^ cfu ml^−1^) in 5 ml microcosms. The three species were combined in a fully factorial design; each species was grown as a monoculture (species richness (δ) = 1, n = 3), with each of the other separately (δ = 2, n = 3) and together (δ = 3, n = 1). Each set of microcosms included a negative (no bacteria) control microcosm and were independently replicated four times (32 microcosms in total). Microcosms were incubated statically for 168 hours with samples taken at 48 and 168 hours.

### Assessment of bacterial growth

The number of colonies of the three bacterial species was recorded at 48 (first time point) and 168 hours (final time point). At each of these times, bacteria were serially diluted in sterile phosphate buffered saline (PBS, pH 7.8, Sigma-Aldrich), following which the samples were inoculated^50^ onto all three selective media and incubated at 37 °C for 48 hours.

### Biological NMR spectroscopy

Supernatant samples were mixed with D_2_O solvent (99% atom%D, Sigma-Aldrich) at a 5:1 ratio. NMR data were collected using a JEOL JNM-ECS400 (JEOL, Tokyo, Japan) NMR spectrometer operating on Delta software (version 5.0.4.5), using a proton resonance frequency of 400 MHz and referenced to the relevant residual solvent peak of water (δ 4.79). Spectra were acquired using a 45° flip angle and a relaxation time of 5 seconds, each spectrum was acquired using 32 scans. ^1^H NMR data were collected using a pre-saturation pulse applied to the water signal to minimise its intensity in the resulting ^1^H NMR spectrum. Spectra were analysed using MestReNova software (version 12.0.4). Integrals were calculated at 0.05 ppm intervals to approximate the abundance of protons at each section of the spectrum.

### Statistical analysis

All statistical analyses and visualisations were performed in R (v3.6.0)^51^ using the vegan (v2.5-4)^52^ and lmerTest (v3.1-0)^53^ packages. All bacterial count data were logarithm (base = 10) transformed prior to analysis and the assumptions for parametric statistics were assessed visually. If these assumptions were not met, non-parametric equivalents were used as stated in the text. In order to account for the repeated sampling of the microcosms a linear mixed effect model (REML = T) was used with “microcosm identity” entered as a random variable. For ^1^H NMR data we used subtraction to highlight differences between the two sampling times (*t*_2_ *– t*_1_). Principle Component Analysis ordinations were calculated using the prcomp() function with correlations assessed using envfit() commands with 999 permutations. All models were simplified in accordance with parsimony with stepwise removal of complex interactions until the simplest significant model remained^54^.

## Supplementary information

Supplementary information.

## Data Availability

Data and R scripts are available on FigShare under the DOIs 10.6084/m9.figshare.11450793.v1 and 10.6084/m9.figshare.11450799.v1 respectively.
