# Supplementary material for: Bacterial dominance is due to effective utilisation of secondary metabolites produced by competitors
Source: Sci Rep. 2020 Feb 11;10:2316. doi: 10.1038/s41598-020-59048-6 (PMC7012823; doi:10.1038/s41598-020-59048-6)
Supplement: Supplementary file 1 — Supplementary information. [file 41598_2020_59048_MOESM1_ESM.pdf]

Title: Bacterial dominance is due to effective utilisation of secondary metabolites produced by competitors.

Authors: Benjamin Morgan, Paul Warren, Ryan E. Mewis & Damian W. Rivett*

Supplementary Figures & Tables

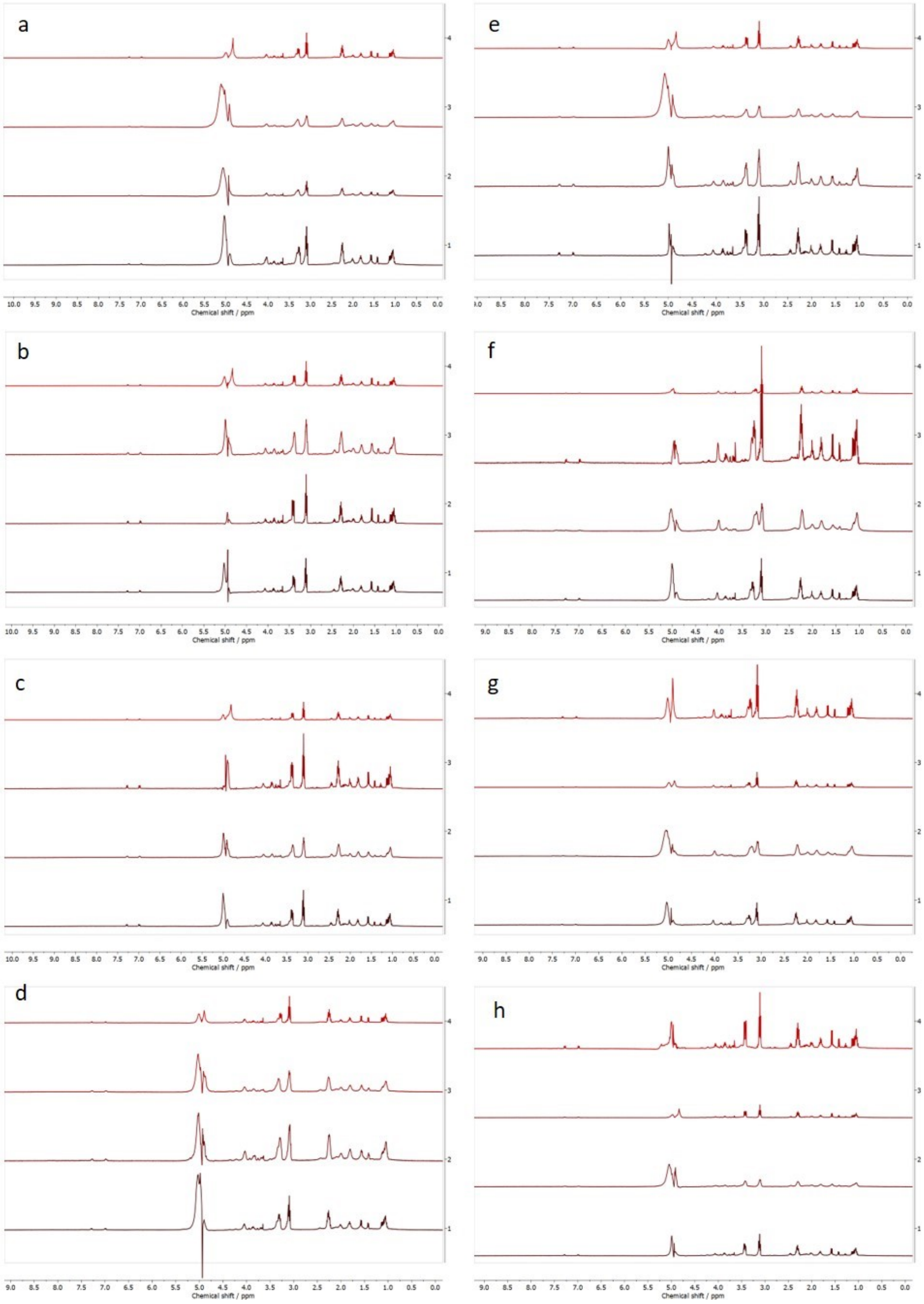

Figure S1: The ^{1}H NMR spectra generated in this study from cell-free media after 48 hours incubation. Each panel represents a different microcosm: a) *Pseudomonas* sp., b) *Burkholderia* sp., c) *Staphylococcus* sp., d) *Burkholderia* sp. & *Pseudomonas* sp., e) *Burkholderia* sp. & *Staphylococcus* sp.,

f) *Pseudomonas* sp. & *Staphylococcus* sp., g) all three isolates together, and h) the uninoculated, sterile control medium.

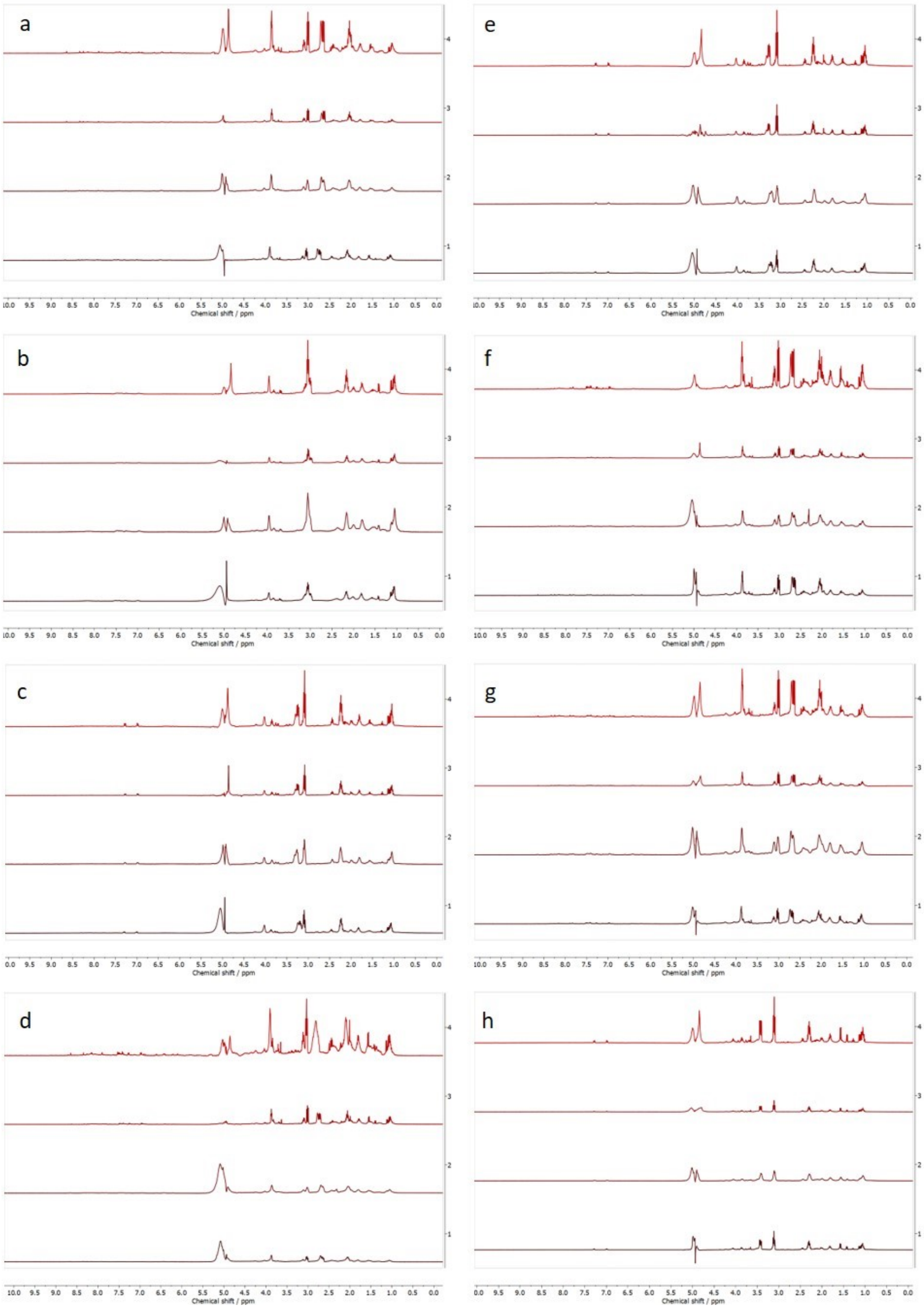

Figure S2: The ^{1}H NMR spectra generated in this study from cell-free media after 168 hours incubation. Each panel represents a different microcosm: a) *Pseudomonas* sp., b) *Burkholderia* sp., c) *Staphylococcus* sp., d) *Burkholderia* sp. & *Pseudomonas* sp., e) *Burkholderia* sp. & *Staphylococcus* sp.,

f) *Pseudomonas* sp. & *Staphylococcus* sp., g) all three isolates together, and h) the uninoculated, sterile control medium.

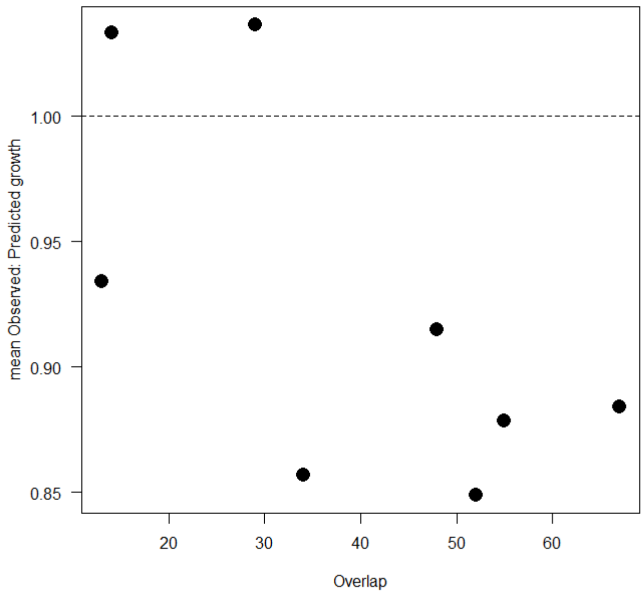

Figure S3: Correlation between niche overlap and mean interaction index (Observed/Predicted growth). In microcosms where the niche overlap between species is lower, a higher than predicted growth is observed compared with those microcosms in which there is a higher overlap.

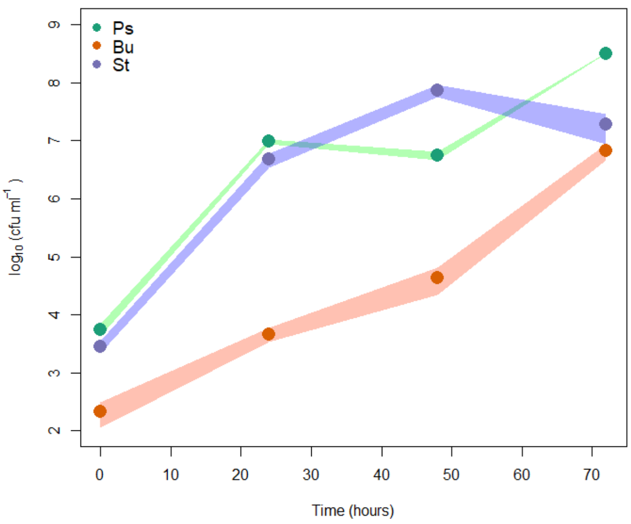

Figure S4: Growth curves of the isolates in the experimental medium.

Table S1:

PCA 1 value	Bacterial initiator	^{1}H NMR peak(s) (ppm)	Corresponding proton	Indicative proton environment
0.180<br>-0.365<br>0.260	Ps<br>BS<br>Ps	2.00 (Arg, Ser)<br>2.20 (Glu, Val)<br>2.70 (Asp)	E	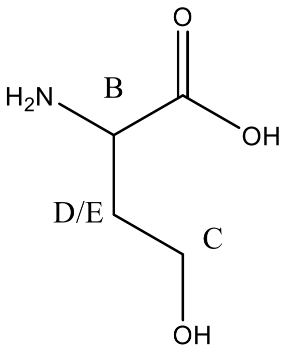
0.186	Ps	2.05 (Pro)	G/F	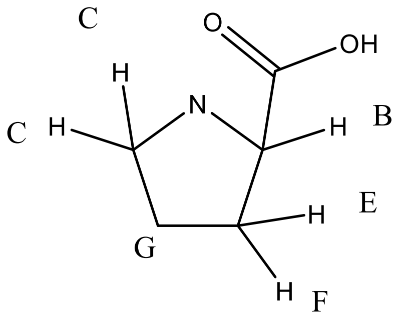
0.303	Ps	2.65 (Met)	C	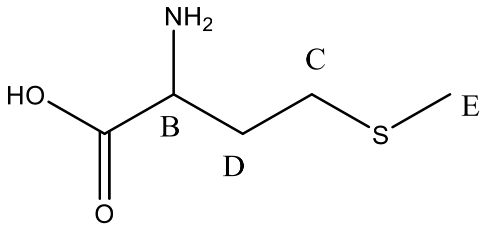
-0.207	BS	3.20 (Cys)	C/D	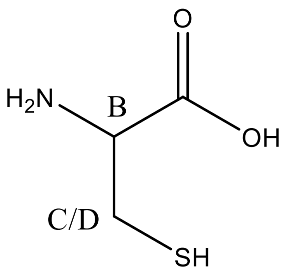

-0.382<br>-0.392<br>-0.180	**BS**	3.05 (Typ, Tyr)<br>3.25 (Phe)<br>3.30 (Typ)	C/J<br>C/J<br>C/J	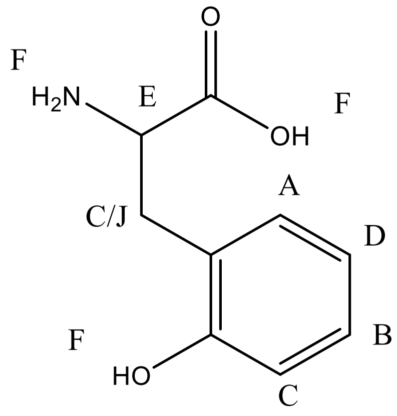
0.265	**Ps**	3.85 (Ser)	B	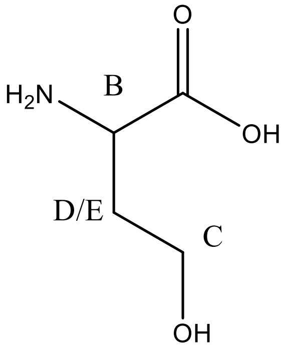
